# Screening and validating HBV mother-to-child transmission-related lncRNAs based on the lncRNA-mRNA co-expression network

**DOI:** 10.3389/fimmu.2026.1829335

**Published:** 2026-06-16

**Authors:** Quan He, Chunyan Zheng, Jiawei Zhang, Yongjian Su, Jialing Li, Shiyi Chen, Haichen Cui, Ting Zeng, Xiong Zou, Hai Li

**Affiliations:** 1 School of Public Health and Management, Guangxi University of Chinese Medicine, Nanning, China; 2 Guangxi Key Laboratory of Translational Medicine for Treating High-Incidence Infectious Diseases with Integrative Medicine, Nanning, China; 3 Beihai People’s Hospital, Nanning, China; 4 GuangDong Second Traditional Chinese Medicine Hospital, Guangzhou, China; 5 Ruikang Hospital Affiliated to Guangxi University of Chinese Medicine, Nanning, China; 6 Zhongshan Hospital of Traditional Chinese Medicine Affiliated to Guangzhou University of Traditional Chinese Medicine, Zhongshan, China; 7 Liuzhou Maternal and Child Health Hospital, Liuzhou, China; 8 The Maternal and Child Health Hospital of Guangxi Zhuang Autonomous Region, Nanning, China

**Keywords:** hepatitis B virus, HRAS, ICAM1, immune regulation, long non-coding RNA, mother-to-child transmission, NAMPT, transcriptomics

## Abstract

**Background:**

Despite standard immunoprophylaxis with hepatitis B immunoglobulin (HBIG) and hepatitis B vaccination, a proportion of infants born to hepatitis B surface antigen (HBsAg)-positive mothers still experience mother-to-child transmission (MTCT) of hepatitis B virus (HBV). The molecular mechanisms underlying immunoprophylaxis failure remain incompletely understood. Long non-coding RNAs (lncRNAs) are increasingly recognized as important regulators of antiviral immune responses; however, their role in HBV MTCT has not been fully elucidated.

**Methods:**

Peripheral blood mononuclear cells (PBMCs) were collected from infants born to HBsAg-positive mothers with either successful or failed MTCT prevention, as well as from healthy controls. Whole-transcriptome RNA sequencing was performed to identify differentially expressed mRNAs and lncRNAs. Gene Ontology (GO) and Kyoto Encyclopedia of Genes and Genomes (KEGG) analyses were conducted to explore the associated biological functions and pathways. An lncRNA–mRNA co-expression network was constructed to identify potential regulatory relationships. Key genes were further validated using real-time quantitative PCR (RT-qPCR).

**Results:**

A total of 2,647 differentially expressed mRNAs and 1,082 differentially expressed lncRNAs were identified between the MTCT prevention failure group and the successful prevention group. Functional enrichment analysis revealed that these genes were mainly involved in immune-related biological processes, including cytokine-mediated signaling, neutrophil activation, and innate immune responses. KEGG pathway analysis demonstrated significant enrichment in pathways related to hepatitis B, MAPK signaling, and NOD-like receptor signaling. The lncRNA–mRNA co-expression network identified 440 potential regulatory interactions associated with MTCT blockade failure. RT-qPCR validation demonstrated that HRAS expression was significantly downregulated in the MTCT blockade failure group, whereas ICAM1, NAMPT, and SOD2 were significantly upregulated compared with healthy controls.

**Conclusion:**

Our findings reveal distinct transcriptomic profiles associated with HBV MTCT and suggest that dysregulated immune-related genes and lncRNA–mRNA regulatory networks may be associated with MTCT prevention failure. In particular, altered expression of HRAS, ICAM1, NAMPT, and SOD2 may represent candidate biomarkers associated with HBV MTCT related to HBV transmission and immune responses in infants. These findings provide preliminary insights into the molecular characteristics of HBV MTCT and may help guide future mechanistic and clinical studies.

## 1 Introduction

Viral hepatitis B (hereinafter referred to as hepatitis B) is an infectious disease caused by hepatitis B virus (HBV) infection and is primarily transmitted through blood exposure (including minor injuries to the skin or mucous membranes) and sexual contact. In China, the prevalence of hepatitis B surface antigen (HBsAg) among pregnant women has declined from approximately 10% in earlier years to 5.4% in 2020; however, the overall infection rate remains relatively high (1).

Although standard intervention strategies, including hepatitis B immunoglobulin (HBIG) and hepatitis B vaccination, have significantly reduced the risk of vertical transmission, these measures cannot completely eliminate MTCT of HBV, and prophylaxis failure still persists (2). Once infected during the neonatal period, more than 90% of infants develop chronic infection. Previous studies have shown that more than 50% of newly diagnosed hepatitis B cases are attributable to intrauterine infection and MTCT (3), and approximately 40%–50% of chronic HBV carriers acquire infection through vertical transmission. Furthermore, about 90% of infants infected with HBV *in utero* eventually develop chronic HBV carrier status. Consequently, vertical transmission has become a major source of new HBV infections and chronic cases (4). Therefore, exploring the mechanisms and influencing factors underlying HBV MTCT, as well as identifying sensitive early biomarkers, is of great importance for the prevention and control of hepatitis B.

Previous studies on the prevention of HBV MTCT have primarily focused on maternal virological and clinical factors, such as hepatitis B viral load, hepatitis B e antigen (HBeAg) status, and antiviral therapy during pregnancy (5). High maternal HBV DNA levels have been consistently identified as a major risk factor for MTCT, even in the context of standard immunoprophylaxis using hepatitis B immunoglobulin (HBIG) and hepatitis B vaccination. Antiviral treatment with agents such as tenofovir disoproxil fumarate in late pregnancy has been shown to further reduce MTCT rates in highly viremic mothers (6).

However, despite these well-established clinical interventions (7), HBV MTCT cannot be completely eliminated, and a proportion of infants still experience prophylaxis failure, suggesting that host-related factors may also play an important role in determining transmission outcomes. Compared with extensive research on maternal and viral factors, relatively limited attention has been paid to the role of infant immune responses and transcriptional regulation in MTCT.

In particular, increasing evidence suggests that non-coding RNAs play important regulatory roles in antiviral immune responses and HBV infection. Among them, microRNAs (miRNAs) have been extensively studied and have been shown to participate in HBV replication, immune escape, and host antiviral responses through post-transcriptional regulatory mechanisms (8, 9). However, compared with miRNAs, long non-coding RNAs (lncRNAs) remain relatively underexplored in the context of HBV MTCT, especially regarding infant immune regulation and prophylaxis failure.

Unlike miRNAs, lncRNAs possess greater regulatory complexity and can function through epigenetic modification, transcriptional regulation, chromatin remodeling, and competing endogenous RNA (ceRNA) mechanisms, thereby influencing multiple downstream immune-related pathways simultaneously (10, 11). Recent studies have shown that lncRNAs can modulate antiviral immune responses and participate in host–virus interactions in chronic HBV infection (12). For example, lnc-AIFM2–1 was reported to promote HBV immune escape by regulating the miR-330-3p/CD244 axis, highlighting the potential importance of lncRNA-mediated regulatory networks in HBV-related immune dysfunction (12). Nevertheless, the specific roles of lncRNAs in HBV MTCT, particularly in infants experiencing immunoprophylaxis failure, remain largely unclear.

Therefore, this study focused on lncRNA-associated transcriptional regulation while acknowledging the important role of miRNAs in HBV infection.

In this study, infants born to HBsAg-positive mothers were recruited from an established cohort, and infants born to HBsAg-negative mothers were included as controls. By performing whole-transcriptome sequencing of PBMC samples, we aimed to identify lncRNA biomarkers associated with HBV MTCT. Furthermore, we constructed an lncRNA–mRNA co-expression network to explore potential regulatory relationships involved in HBV MTCT. By focusing on peripheral blood mononuclear cells (PBMCs) of infants, this study seeks to provide preliminary evidence regarding the role of lncRNA–mRNA transcriptional regulation in HBV MTCT and to offer a scientific basis for improving prevention and control strategies for HBV MTCT.

## 2 Materials and methods

### 2.1 Sample acquisition

All experimental procedures in this study were approved by the Ethics Committee of Guangxi University of Chinese Medicine(ref: GXUCM IRB H 2023-06-01-1). Researchers retrieved prenatal examination information of pregnant women who gave birth in hospitals and maternal and child health care institutions at all levels in Liuzhou City from July 1, 2023 to January 3, 2024, from the “Gui Women and Children’s Health Service Information Management System”, and provided intervention services to prevent MTCT of hepatitis B for mothers who were positive for HBsAg during prenatal examinations. Peripheral venous blood (7 mL) was collected from infants aged 7–24 months who had received hepatitis B immunoglobulin (HBIG) within 24 hours after birth and had completed the three-dose hepatitis B vaccination schedule according to the national immunization program 1–2 months prior to sampling. Among them, 5 ml was used for hepatitis B serological testing, and 2 ml was used to extract RNA for subsequent sequencing.

**Figure 1:**
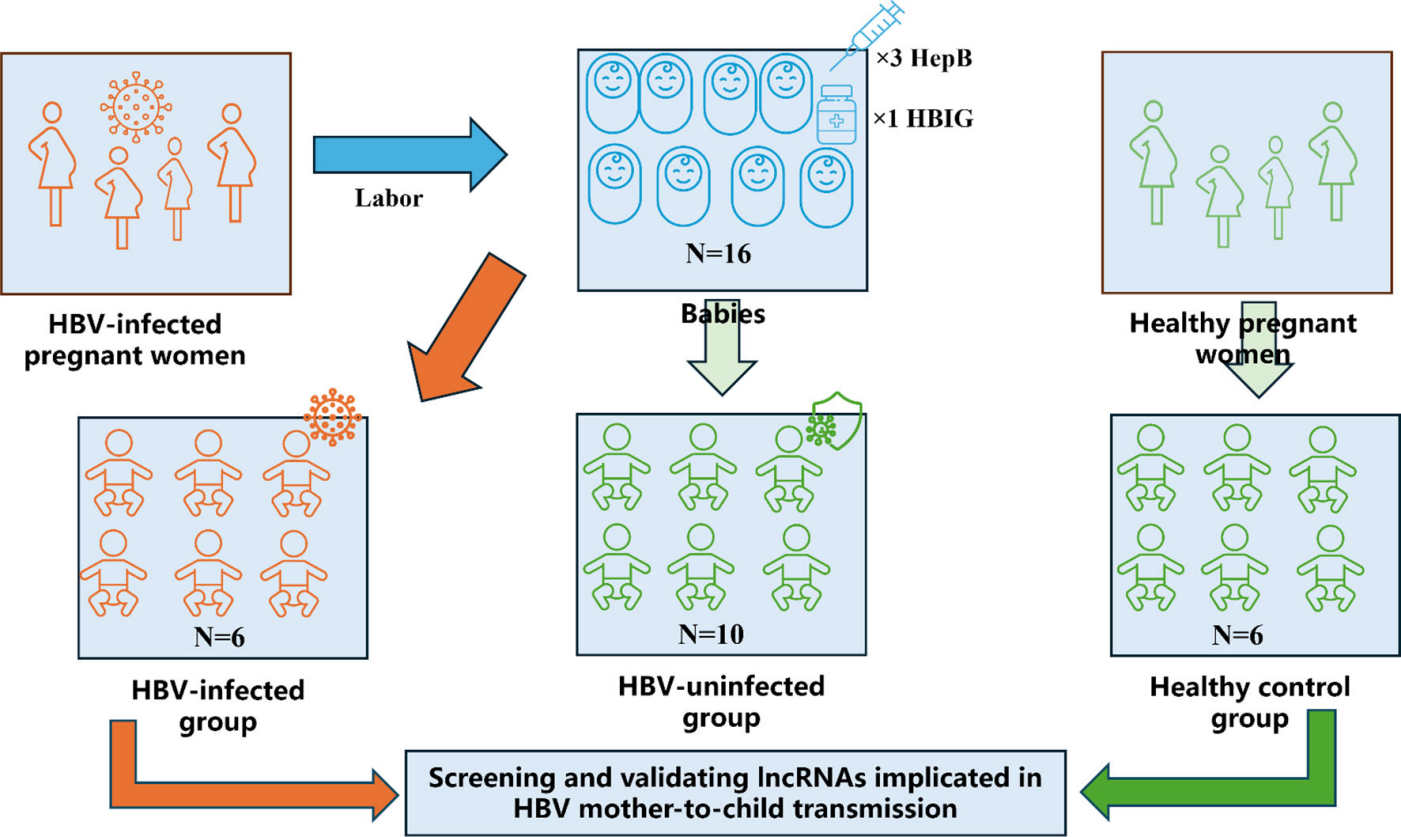
Chart of study group allocation.

A total of 133 venous blood samples from infants born to HBsAg-positive mothers and 6 venous blood samples from infants born to HBsAg-negative mothers were obtained in this study. According to the results of hepatitis B serological tests, the HBsAg-positive infants were divided into the “HBV mother-to-child blockade failure group(HBV-infected group)”, and the HBsAg-negative infants were divided into the “HBV mother-to-child blockade success group(HBV-uninfected group)”. Meanwhile, healthy infants who came for physical examinations were recruited from the Department of Child Health Care in Liuzhou Maternity and Child Health Care Hospital as the healthy control group. Details are shown in **Figure 1**. After quality control and eligibility screening, 22 samples were selected for transcriptome sequencing, including 6 MTCT failure cases, 10 MTCT success cases, and 6 healthy controls.

Grouping was carried out based on the test results of hepatitis B serum markers and the HBV DNA load. According to the *Information Collection Form for HBsAg-Positive Pregnant and Postpartum Women and Their Infants* collected during the follow-up period, the general demographic characteristics of the mothers, pregnancy and antenatal examination data, results of hepatitis B serum marker examinations during pregnancy, antiviral treatment and other situations were obtained. Based on the “Follow-Up Card for Infants Born to HBsAg-Positive Pregnant and Postpartum Women”, information such as the general demographic characteristics, birth conditions, and HepB vaccination status of the infants was acquired to match the case group with the control group.

In the case group and the first control group, the mothers’ hepatitis B serological tests during prenatal examinations all showed positive results for HBsAg, HBeAg, and hepatitis B core antibody (Hepatitis B surface antibody, anti-HBc), which is commonly known as “big three positive”. All the infants were normal-weight full-term infants at birth. All of them had completed the inoculation of three doses of HepB (active immunization) and had been injected with one dose of 100 international units of HBIG (passive immunization) after birth. In the group with failed MTCT blocking of HBV, there were 3 males and 3 females respectively, while in the group with successful MTCT blocking of HBV, there were 5 males and 5 females respectively. There was no difference in the gender composition ratio between the two groups of research subjects.

There were no statistically significant differences in maternal age or delivery mode between the case group and the first control group (FDR-adjusted p value > 0.05). Additionally, no significant differences were observed in maternal HBV DNA levels during prenatal examinations or in antiviral treatment status between the two groups (FDR-adjusted p value > 0.05) (**Table 1**).

**Table 1:** Comparison of the basic information of the two groups of research subjects.

Variable	HBV mother-to-fetal blockade failure group *n* = 6	HBV mother-to-child blockade success group *n* = 10	*FDR-adjusted p value*
Mother’s age	31.67 ± 2.58	30.70 ± 5.21	0.680
HBVDNA test for maternal prenatal checkups(*n,%*)	1.87×10^8^ ± 2.32×10^8^	1.27×10^3^(1.00×10^2^, 3.35×10^5^)	0.423
Maternal antiviral therapy(*n,%*)			
No Medication	4(60)	5(50)	0.515
Medication	2(40)	5(50)	
Mode of delivery (*n*,%)			
Natural childbirth	4(66.67)	8(80)	0.837
Emergency cesarean section	1(16.67)	1(10)	
Elective cesarean section	1(16.67)	1(10)	
Infants and young children were followed up with hepatitis B serology test results(n)			
HBsAg, HBeAg and HBcAb test positive	1	/	
HBsAg, HBeAb and HBcAb test positive	5		
Quantitative results of HBV DNA in infants and young children were followed-up(n)			
≥2×10^2^ IU/mL	6	/	
<2×10^2^ IU/mL	0		

To reduce potential confounding effects, efforts were made to ensure comparability between groups. All infants included in the study were full-term with normal birth weight and had received standardized immunoprophylaxis, including HBIG administration within 24 hours after birth and completion of the three-dose hepatitis B vaccination schedule. In addition, mothers in both the MTCT failure and success groups were HBsAg-positive and HBeAg-positive, which helped minimize variability related to maternal infection status.

However, due to the retrospective and exploratory nature of the study, not all potential influencing factors, such as dynamic HBV DNA levels, timing of antiviral therapy, and detailed immune parameters, could be fully controlled or included in the analysis.

### 2.2 RNA extraction and quality control

Collect 2 mL of peripheral venous blood from the research subjects and extract and purify the total RNA using the Qiagen PAXgene Blood RNA Kit. To ensure the construction of high-quality total RNA-seq libraries, the Invitrogen Qubit 3.0 Fluorometer was used to evaluate the concentration of the extracted total RNA; the NanoDrop 2000 spectrophotometer was used to measure RNA purity and concentration; RNA integrity was assessed using agarose gel electrophoresis; and the Agilent 2100 Bioanalyzer was used to assess RNA quality.

### 2.3 Library construction and RNA sequencing

A total of 6 samples from the HBV MTCT blockade failure group, 10 samples from the HBV MTCT blockade success group, and 6 samples from the healthy control group, all of which met the quality control standards, were selected for library construction. Sequencing was carried out using the Illumina HiSeq/NovaSeq platform (Illumina, USA) by Shanghai Tianhao Biotechnology Co., Ltd. The libraries were sequenced using a paired-end strategy with 150 bp paired-end reads.

The raw reads were filtered using FastQC and R software to obtain clean reads for downstream analysis. The filtered reads were aligned to the human genome (GRCh38) using STAR software (13, 14), and the results of the STAR alignment were statistically analyzed using Picard. The quality of the Total RNA library was evaluated using the RSeQC software (15). The analysis pipeline of Stringtie was utilized to perform expression quantification on known genes and transcripts (16), obtaining fragments per kilobase of transcript per million mapped reads (FPKM) at the gene level. To detect potential outliers or deviation values, principal component analysis (PCA) was carried out using the Factoextra R package. The Pearson correlation coefficient was used to analyze the correlations between samples, and the results were visualized through a heatmap.

### 2.4 Differential expression analysis and enrichment analysis of mRNA and lncRNA

The analysis of differentially expressed mRNAs and lncRNAs was performed using DESeq2 (17, 18). Comparisons were made between the HBV MTCT blocking failure group and the HBV MTCT blocking success group (A vs B), as well as between the HBV MTCT blocking failure group and the healthy control group (A vs C). After Benjamini–Hochberg multiple testing correction, genes with FDR-adjusted p value< 0.05 and an absolute log2 fold change > 1 were considered differentially expressed genes (DEGs). The results were visualized using bar charts, volcano plots, and clustered heatmaps.

The Gene Ontology (GO) database established by the Gene Ontology Consortium can classify genes with significant differences according to molecular function, cellular component, and biological process categories (19). GO functional enrichment analysis allows visualization of the distribution patterns of differentially expressed genes and helps reveal functional differences among experimental samples.

Kyoto Encyclopedia of Genes and Genomes (KEGG) pathway enrichment analysis was used to identify the major biochemical metabolic pathways and signaling pathways associated with the differentially expressed genes (20).

GO and KEGG enrichment analyses of the significantly differentially expressed genes were performed using the clusterProfiler package in R to infer the potential molecular mechanisms underlying HBV MTCT (21).

### 2.5 Analysis of lncRNA-mRNA co-expression network

The co-expression analysis is based on the following two points: (1) The expression levels of lncRNA/mRNA show significant differences between groups; (2) There is a targeting relationship between lncRNA and mRNA. The cor.test function in the R language is used to conduct a correlation test on the expression levels of differential lncRNAs and differential mRNAs. The lncRNA-mRNA co-expression network is constructed according to the condition that the FDR-adjusted p value <0.05, and the co-expression network of lncRNA and mRNA is presented through Cytoscape 3.7.1 (22).

Furthermore, GO and KEGG enrichment analyses were performed on the mRNAs involved in the lncRNA–mRNA co-expression network to further explore the potential biological functions and signaling pathways associated with HBV MTCT. In addition, a protein–protein interaction (PPI) network analysis of the differentially expressed mRNAs in the co-expression network was conducted using the STRING database and visualized using Cytoscape software.

### 2.6 Validation of differentially expressed genes by RT-qPCR

The hub genes related to HBV MTCT were identified by using the MCODE and cytohubba plugins in Cytoscape. Combined with the key pathways analyzed by KEGG clustering, ten differentially expressed genes were selected for RT-qPCR validation.

Using GAPDH as an internal reference control, the SYBR^®^ Premix Ex Taq™ II fluorescent dye reagent was employed for amplification on the Roche LightCycler 480II real-time fluorescent quantitative PCR instrument. The relative quantification of the target genes was calculated and analyzed by the 2-ΔCp method (23). All the detections were repeated three times.

### 2.7 Statistical analysis

Data statistical analysis was performed using SPSS 20.0 software. The measurement data conforming to the normal distribution were described by mean ± standard deviation. For the data with normal distribution and homogeneous variance, the t-test was used for the comparison between two groups. Since the sample size in this article<40, the Fisher’s exact χ^2^ test method was adopted when comparing categorical variables between groups. The two-sided test with α = 0.05 was selected for all the above test methods. The criteria for screening differentially expressed genes (DEGs) were FDR-adjusted p value < 0.05 and |log2 FC| ≥ 1.

## 3 Results

### 3.1 Analysis of differential gene expression

**Figure 2:**
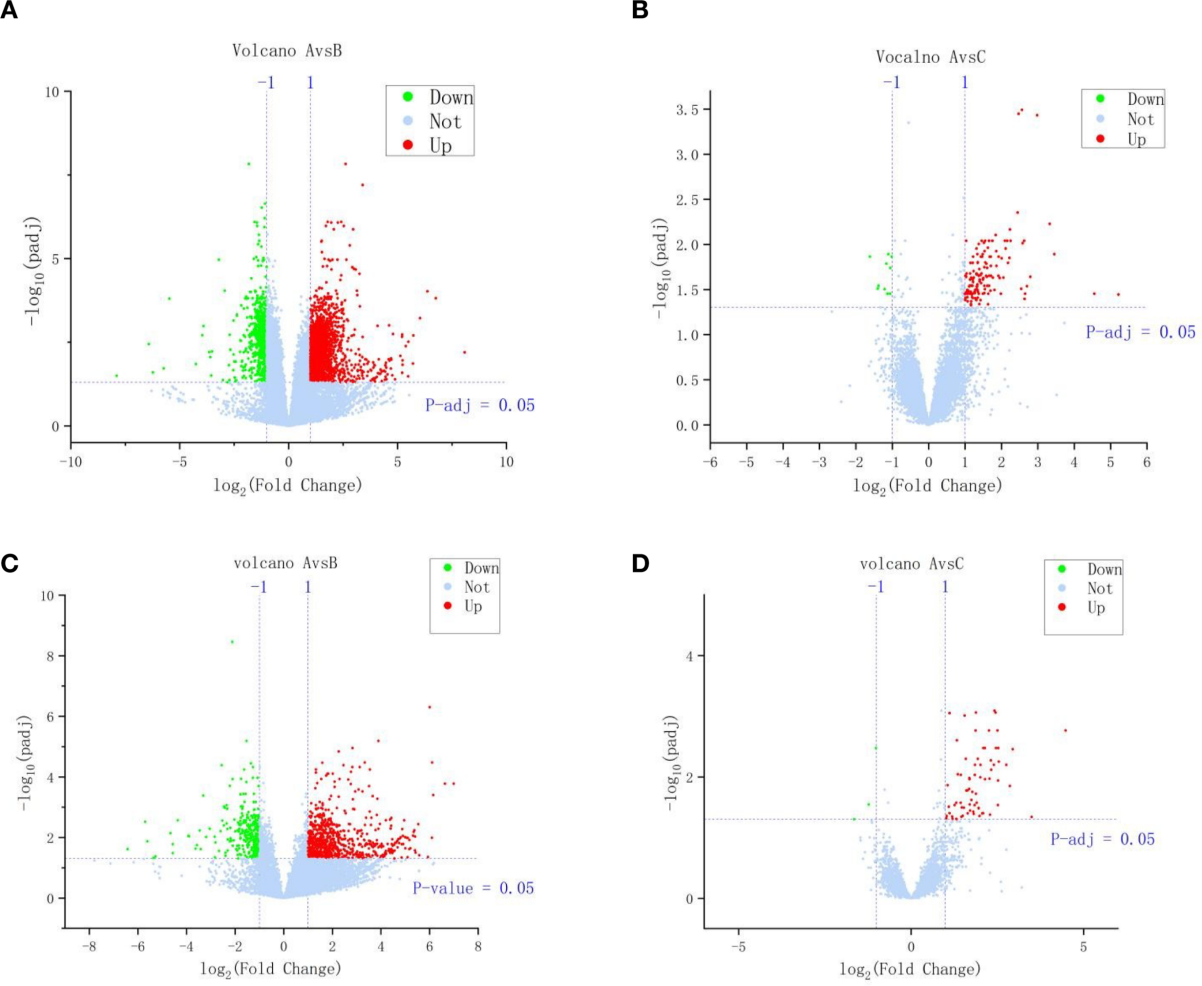
Analysis of differentially expressed mRNAs. **(A)** Volcano plot of differentially expressed mRNAs in the HBV mother-to-child transmission blocking failure group vs the HBV mother-to-child transmission blocking success group. **(B)** Volcano plot of differentially expressed mRNAs in the HBV mother-to-child transmission blocking failure group vs the HBV healthy control group. **(C)** Clustered heatmap of mRNAs with significantly differential expressions in the HBV mother-to-child transmission blocking failure group vs the HBV mother-to-child transmission blocking success group. **(D)** Clustered heatmap of mRNAs with significantly differential expressions in the HBV mother-to-child transmission blocking failure group vs the HBV healthy control group.

Compared with the HBV MTCT blocking success group, 2,647 differentially expressed mRNAs (DEGs) were identified in the HBV MTCT blocking failure group, including 1,793 up-regulated and 854 down-regulated genes (**Figure 2A**). Representative up-regulated genes included CD177, SUCNR1, and BMX, whereas representative down-regulated genes included KRT19, SOX9, and PCDH7 (**Figure 2A**).

Compared with the healthy control group, 151 DEGs were identified in the HBV MTCT blocking failure group, including 141 up-regulated and 10 down-regulated genes (**Figure 2B**). Representative up-regulated genes included CD177, FFAR3, and PROK2, whereas NBPF3 and LRP6 were among the down-regulated genes (**Figure 2B**).

A total of 28 commonly up-regulated mRNAs and one commonly down-regulated mRNA were identified across comparisons. Commonly up-regulated genes included CD177, PROK2, IL1R1, and TREM1, suggesting shared immune-related transcriptional alterations associated with HBV MTCT blockade failure.

Heatmap analysis demonstrated distinct mRNA expression patterns between groups, with several inflammatory and immune-related genes showing opposite clustering trends between the HBV MTCT blocking failure and control groups (**Figures 2C, D**).

**Figure 3:**
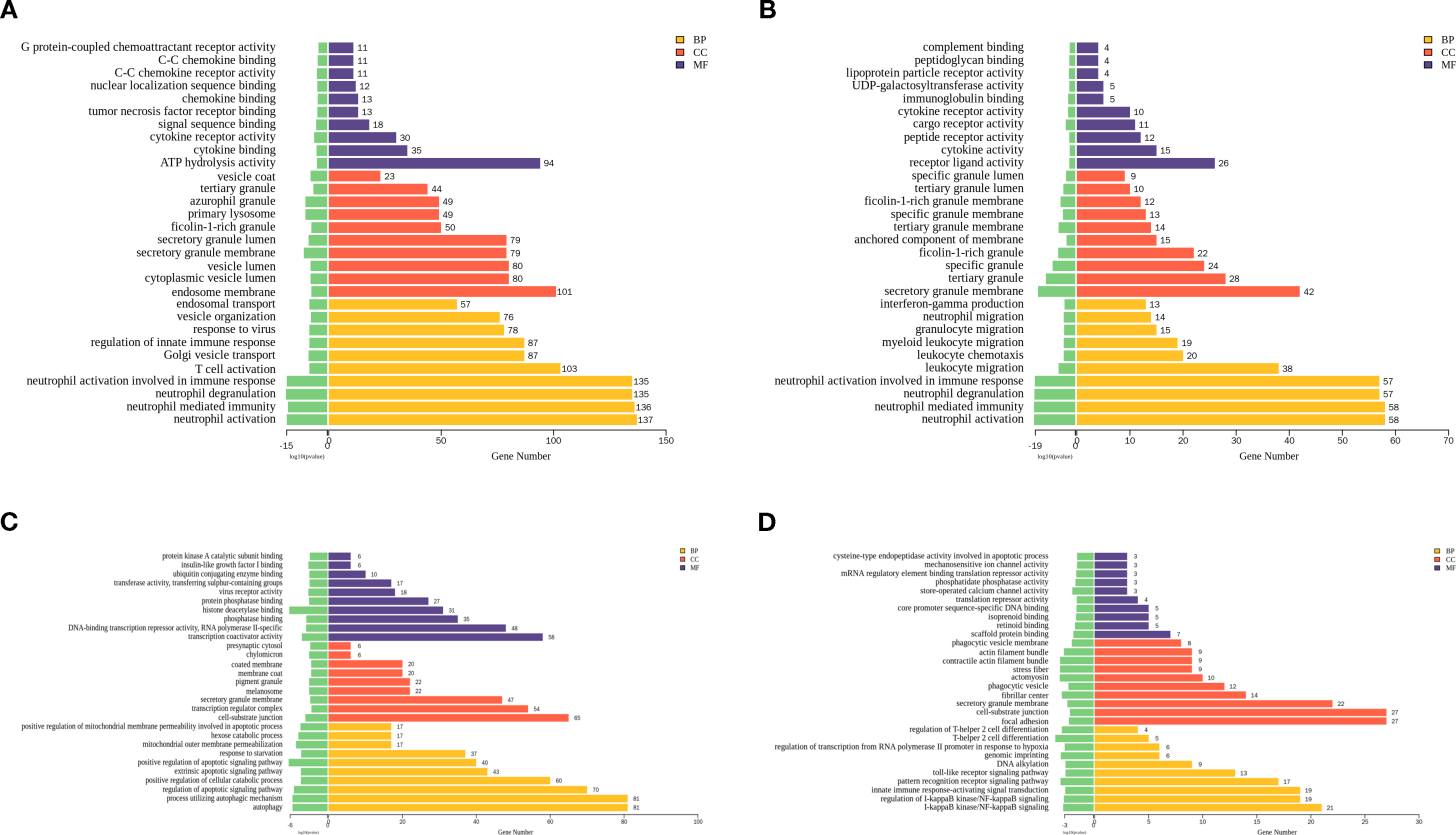
Analysis of differentially expressed lncRNAs. **(A)** Volcano plot of differentially expressed lncRNAs in the group with failed HBV mother-to-child transmission blockage vs the group with successful HBV mother-to-child transmission blockage. **(B)** Volcano plot of differentially expressed lncRNAs in the group with failed HBV mother-to-child transmission blockage vs the HBV healthy control group. **(C)** Cluster heat map of significantly differentially expressed lncRNAs in the group with failed HBV mother-to-child transmission blockage vs the group with successful HBV mother-to-child transmission blockage. **(D)** Cluster heat map of significantly differentially expressed lncRNAs in the group with failed HBV mother-to-child transmission blockage vs the HBV healthy control group.

Compared with the HBV MTCT blocking success group, 1,082 differentially expressed lncRNAs were identified in the HBV MTCT blocking failure group, including 755 up-regulated and 327 down-regulated lncRNAs (**Figure 3A**).

Compared with the healthy control group, 75 differentially expressed lncRNAs were identified, including 72 up-regulated and 3 down-regulated lncRNAs (**Figure 3B**).

Thirty-one commonly up-regulated lncRNAs were identified across comparisons, including NEAT1 and BASP1-AS1, whereas no commonly down-regulated lncRNAs were observed.

Heatmap analysis revealed distinct clustering patterns of lncRNA expression between groups, indicating substantial transcriptional differences associated with HBV MTCT blockade failure (**Figures 3C, D**).

### 3.2 GO enrichment analysis of mRNA and lncRNA

**Figure 4:**
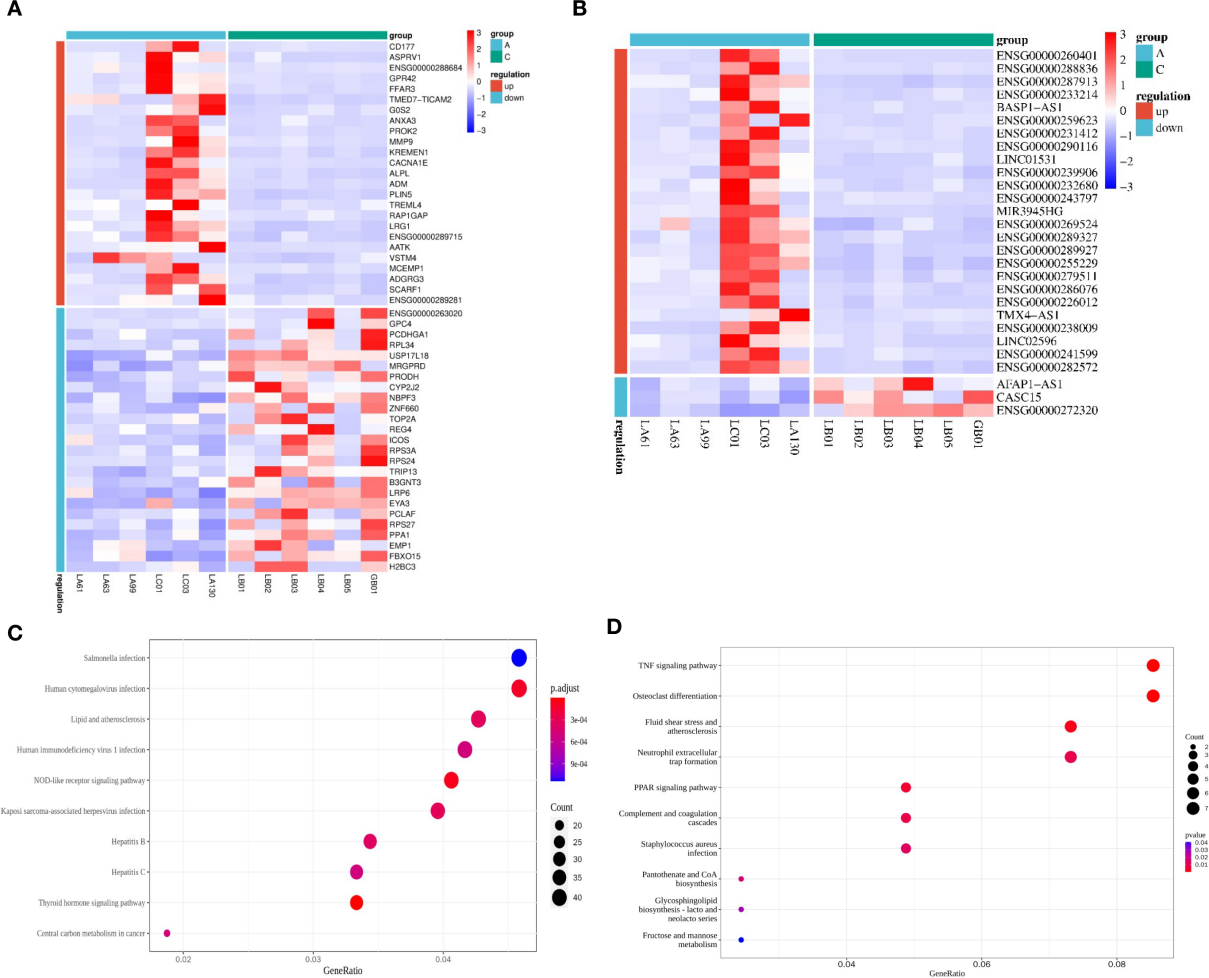
GO clustering analysis of differentially expressed mRNAs and lncRNAs. **(A)** GO Enrichment Bar Chart of Significantly Differentially Expressed mRNAs in the Group with Failed HBV Mother-to-Child Transmission Blockage vs the Group with Successful HBV Mother-to-Child Transmission Blockage. **(B)** GO Enrichment Bar Chart of Significantly Differentially Expressed mRNAs in the Group with Failed HBV Mother-to-Child Transmission Blockage vs the Healthy Control Group. **(C)** GO Enrichment Bar Chart of Significantly Differentially Expressed lncRNAs in the Group with Failed HBV Mother-to-Child Transmission Blockage vs the Group with Successful HBV Mother-to-Child Transmission Blockage. **(D)** GO Enrichment Bar Chart of Significantly Differentially Expressed lncRNAs in the Group with Failed HBV Mother-to-Child Transmission Blockage vs the Healthy Control Group.

GO enrichment analysis showed that differentially expressed mRNAs between the HBV MTCT blocking failure and success groups were mainly enriched in immune-related biological processes, including neutrophil activation, innate immune regulation, antiviral response, and vesicle-mediated transport (**Figure 4A**).

Compared with healthy controls, differentially expressed mRNAs were primarily enriched in neutrophil-mediated immunity, inflammatory responses, cytokine production, and bacterial response-related pathways (**Figure 4B**).

Differentially expressed lncRNAs were mainly enriched in transcriptional regulation, apoptosis signaling, autophagy, focal adhesion, and immune-related pathways (**Figures 4C, D**).

### 3.3 KEGG enrichment analysis of mRNA and lncRNA

**Figure 5:**
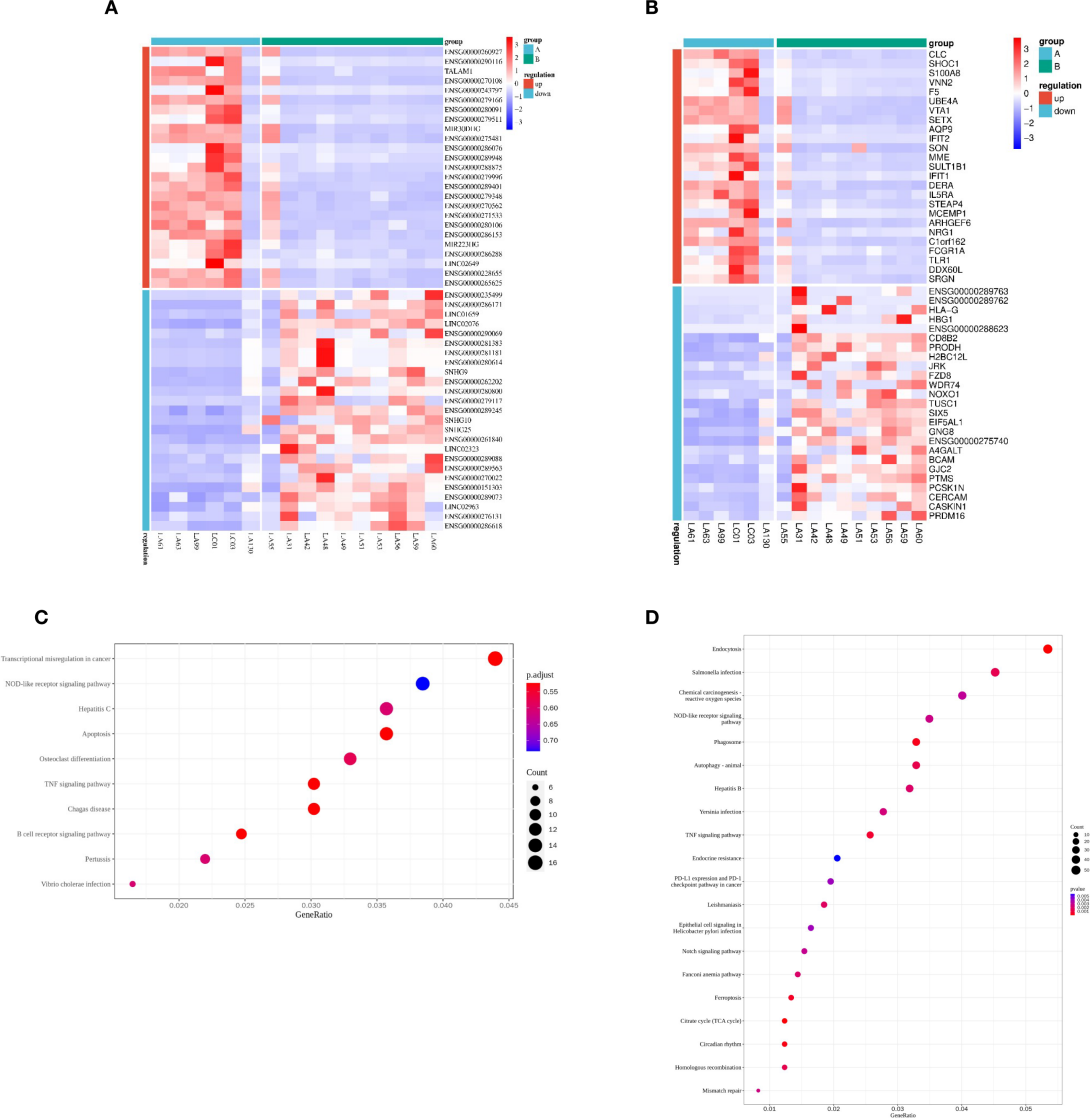
KEGG clustering analysis of differentially expressed mRNAs and lncRNAs. **(A)** KEGG Enrichment Scatter Plot of Significantly Differentially Expressed mRNAs in the Group with Failed HBV Mother-to-Child Transmission Blockage vs the Group with Successful HBV Mother-to-Child Transmission Blockage. **(B)** KEGG Enrichment Scatter Plot of Significantly Differentially Expressed mRNAs in the Group with Failed HBV Mother-to-Child Transmission Blockage vs the Healthy Control Group. **(C)** KEGG Enrichment Scatter Plot of Significantly Differentially Expressed lncRNAs in the Group with Failed HBV Mother-to-Child Transmission Blockage vs the Group with Successful HBV Mother-to-Child Transmission Blockage. **(D)** KEGG Enrichment Scatter Plot of Significantly Differentially Expressed lncRNAs in the Group with Failed HBV Mother-to-Child Transmission Blockage vs the Healthy Control Group.

KEGG analysis demonstrated that differentially expressed mRNAs were mainly enriched in immune- and virus-related pathways, including the NOD-like receptor signaling pathway, hepatitis B pathway, autophagy, and endocytosis (**Figure 5A**). Compared with healthy controls, enriched pathways mainly involved inflammatory and immune regulation, including TNF signaling and neutrophil extracellular trap formation (**Figure 5B**).

Target genes of differentially expressed lncRNAs were enriched in multiple immune-related signaling pathways, including the NOD-like receptor, MAPK, PI3K-Akt, and hepatitis-related pathways (**Figures 5C, D**).

### 3.4 Analysis of mRNA-lncRNA co-expression network

Based on the mRNA and lncRNA differential expression results in Section 2.2, an lncRNA–mRNA cis-regulatory co-expression network was constructed. The cor.test function was used to perform correlation analysis between differentially expressed lncRNAs and mRNAs. Using FDR-adjusted p value < 0.05 as the screening criterion, a positive correlation (cor > 0) indicated positive co-expression, whereas a negative correlation (cor < 0) indicated inverse expression patterns.

**Figure 6:**
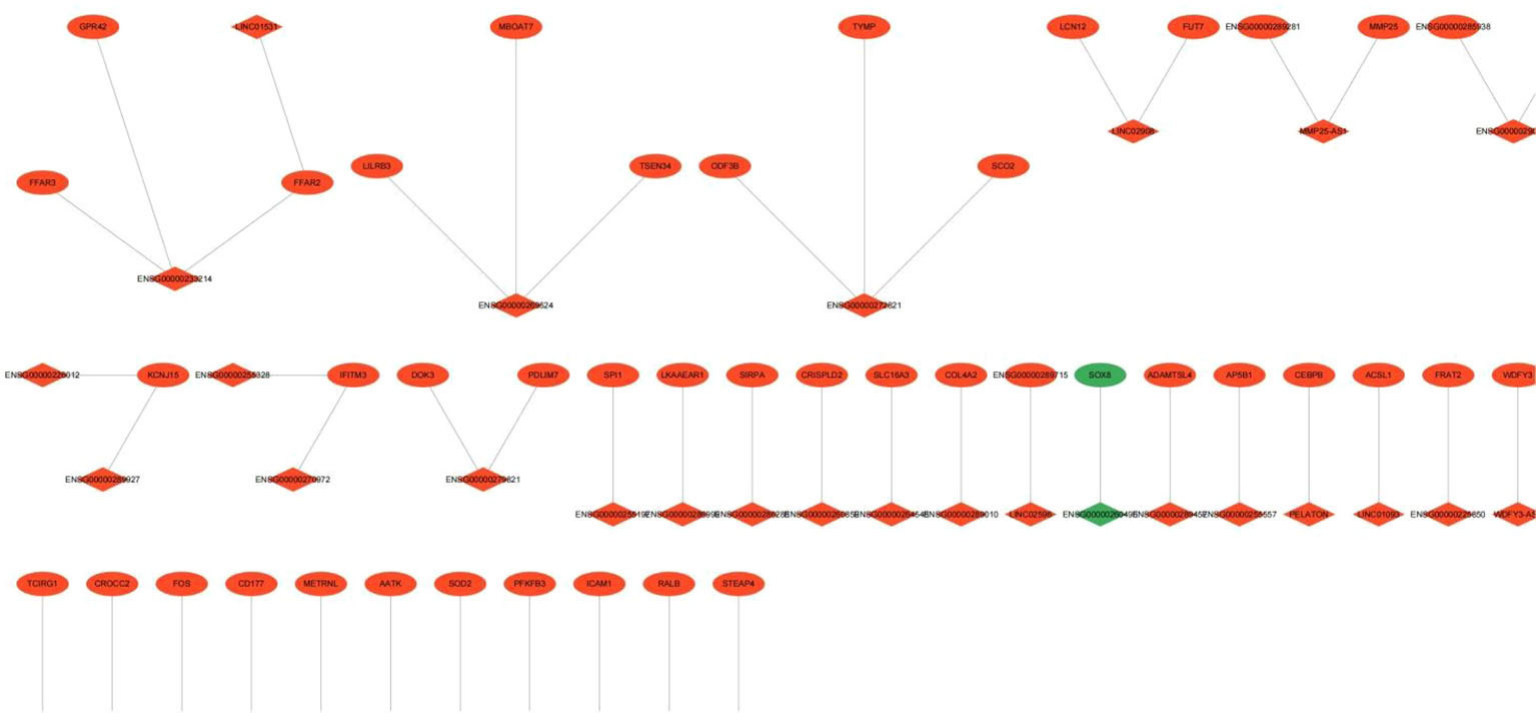
Network diagram of lncRNA - mRNA Cis-regulatory interactions in the group with failed HBV mother-to-child transmission blockage vs the group with successful HBV mother-to-child transmission blockage.

**Figure 7:**
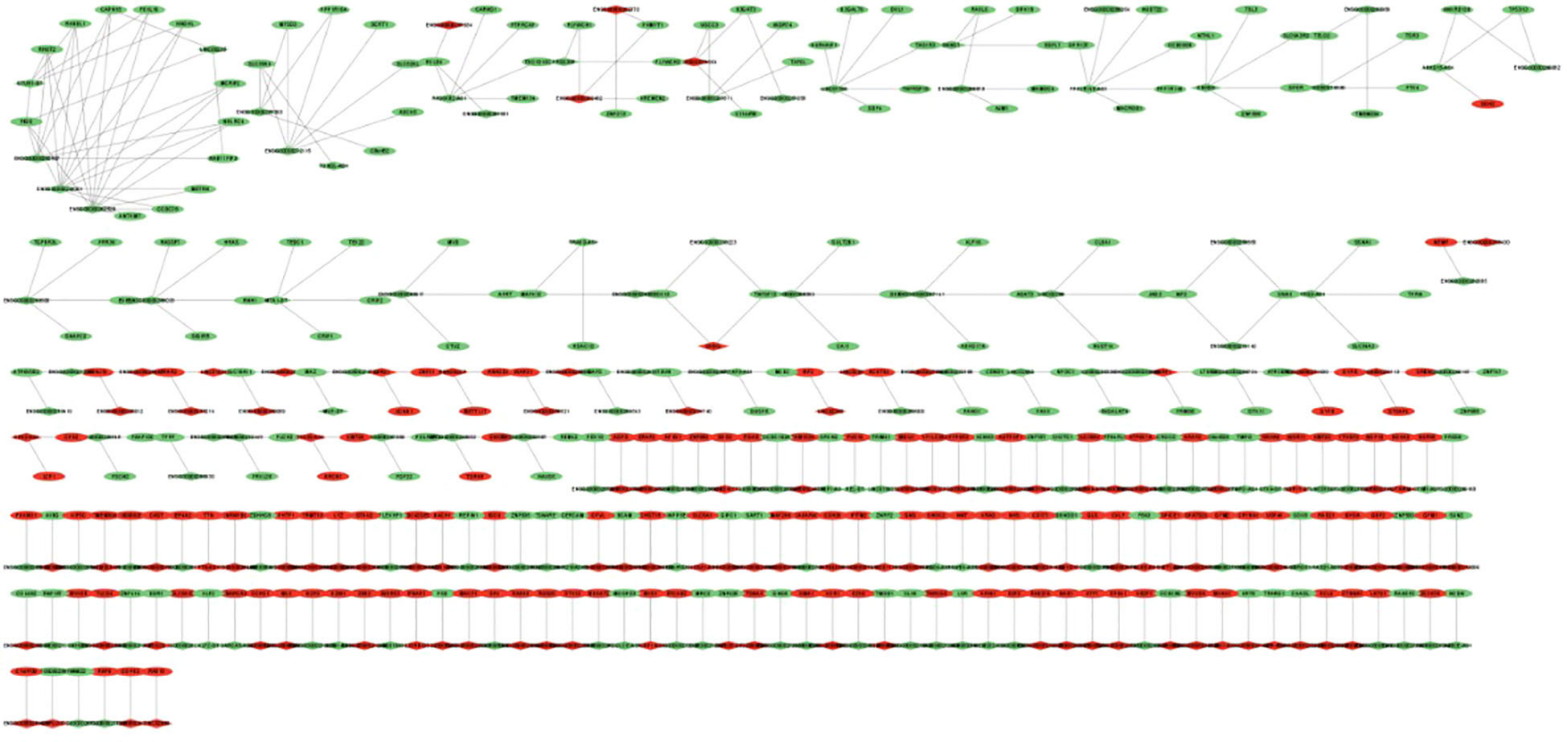
Network diagram of lncRNA - mRNA Cis-regulatory interactions in the group with successful HBV mother-to-child transmission blockage vs the healthy control group. The oval represents mRNA, and the diamond represents lncRNA. Red indicates that the RNA is up-regulated in expression, while green indicates that the RNA is down-regulated in expression.

A total of 440 lncRNA–mRNA co-expressed gene pairs were identified between the HBV MTCT blocking failure group and the HBV MTCT blocking success group, and 72 co-expressed gene pairs were identified between the HBV MTCT blocking failure group and the healthy control group (**Table 2**). Among them, ENSG00000228201 regulated 11 mRNAs, ENSG00000262528 regulated 11 mRNAs, and lncRNA ENSG00000282907 regulated 7 mRNAs (**Figures 6**, **7**).

**Table 2:** Statistical table of interactions between lncRNAs and mRNAs.

Group_1_vs_group_2	lncRNA up-up mRNA	lncRNA up-down mRNA	lncRNA down-up mRNA	lncRNA down-down mRNA
A_vs_B	101	59	55	225
A_vs_C	68	/	2	2

#### 3.4.1 GO and KEGG enrichment analysis of mRNA-lncRNA co-expression network

**Figure 8:**
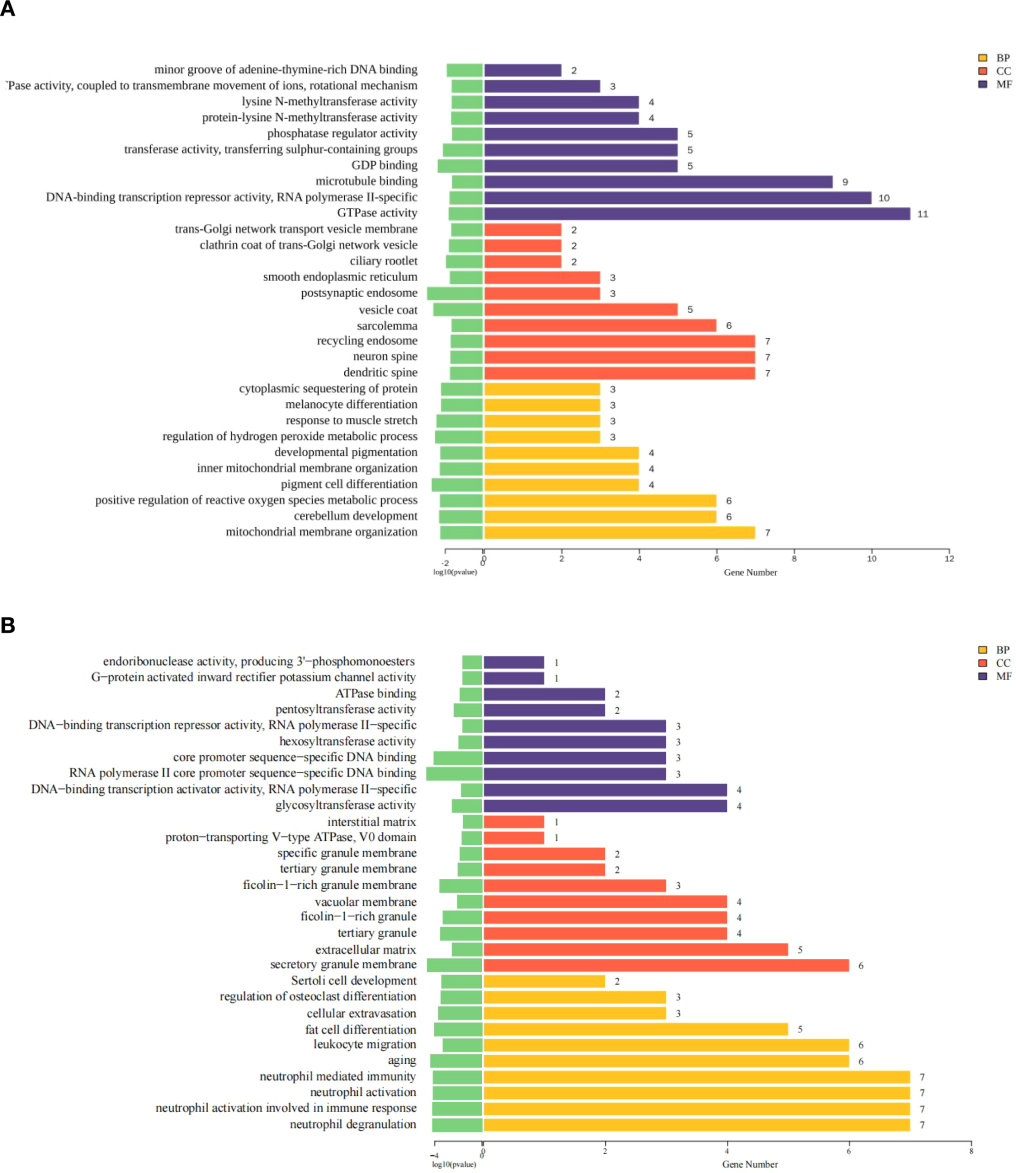
**(A)** GO enrichment bar chart showing significant differences in mRNA lncRNA co-expression between the group with failed HBV mother-to-child transmission blockage vs the group with successful HBV mother-to-child transmission blockage. **(B)** GO enrichment bar chart showing significant differences in mRNA lncRNA co-expression between the group with failed HBV mother-to-child transmission blockage vs the healthy control group.

**Figure 9:**
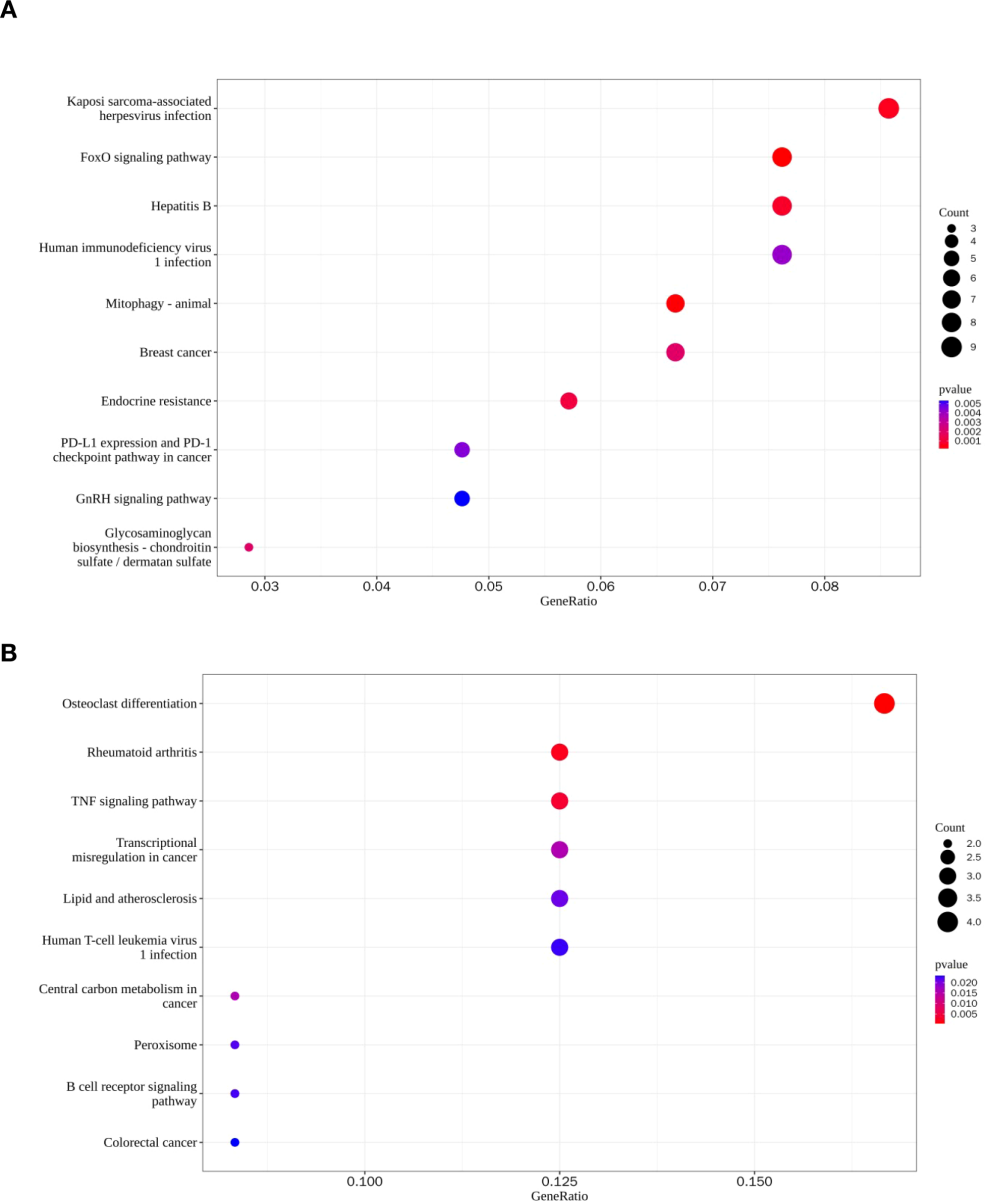
**(A)** KEGG enrichment scatter plot of significant differences in mRNA lncRNA co -expression between the group with failed HBV mother-to-child transmission blockage and successful HBV mother-to-child transmission blockage. **(B)** KEGG enrichment scatter plot of significant differences in mRNA lncRNA co-expression between the group with failed HBV mother-to-child transmission blockage and the healthy control group.

Functional enrichment analysis of the lncRNA–mRNA co-expression network indicated that the co-expressed genes were mainly involved in immune regulation, inflammatory responses, transcriptional regulation, and HBV-related signaling pathways. KEGG analysis further identified significant enrichment in hepatitis B, NOD-like receptor, TNF, and FoxO signaling pathways, suggesting potential involvement of these networks in antiviral immune responses during HBV MTCT (**Figures 8**, **9**).

#### 3.4.2 Protein–protein interaction network analysis of the lncRNA–mRNA co-expression network

**Figure 10:**
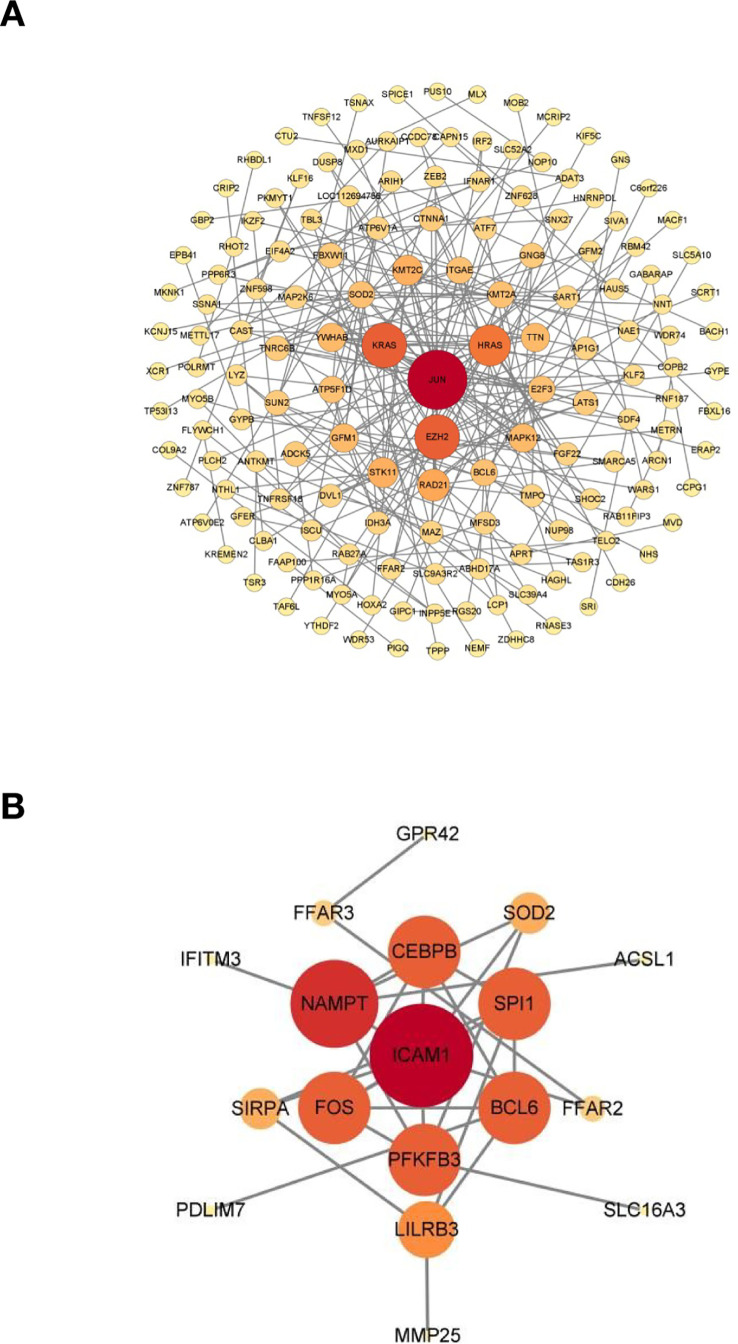
**(A)** PPI regulatory interaction network of lncRNA mRNA co-expression differences between the group with failed HBV mother-to-child transmission blockage and successful HBV mother-to-child transmission blockage. **(B)** PPI regulatory interaction network of lncRNA mRNA co-expression differences between the group with failed HBV mother-to-child transmission blockage and the healthy control group.

In the comparison between the HBV MTCT blockade failure group and the HBV MTCT blockade success group, the PPI network demonstrated that JUN occupied a relatively central position in the interaction network, suggesting potential involvement in immune regulation and intracellular signaling processes associated with HBV MTCT (**Figure 10A**). Using the cytoHubba plugin, the following hub genes were identified: JUN, KMT2C, KRAS, HRAS, STK11, E2F3, and BCL6. These genes were mainly associated with transcriptional regulation, antiviral immune responses, intracellular signaling pathways, and cell proliferation-related processes.

In the comparison between the HBV MTCT blockade failure group and the healthy control group, ICAM1 was located at the center of the PPI network and exhibited extensive interactions with other differentially expressed genes (**Figure 10B**). Based on the MCODE algorithm, the major hub genes identified included ICAM1, NAMPT, FOS, PFKFB3, BCL6, SPI1, and CEBPB. These genes were primarily enriched in inflammatory activation, cytokine-mediated signaling, immune-cell differentiation, and immunometabolic regulation pathways, suggesting that dysregulated immune and inflammatory responses may participate in the mechanisms underlying HBV MTCT blockade failure.

Overall, the PPI network analysis further supported the potential importance of JUN, HRAS, ICAM1, NAMPT, and other hub genes in antiviral immune regulation, inflammatory responses, and HBV-related signaling pathways during HBV MTCT. These hub genes may serve as candidate biomarkers or potential molecular targets for future mechanistic studies.

#### 3.4.3 Screening of genes based on lncRNA-mRNA co-expression network

**Figure 11:**
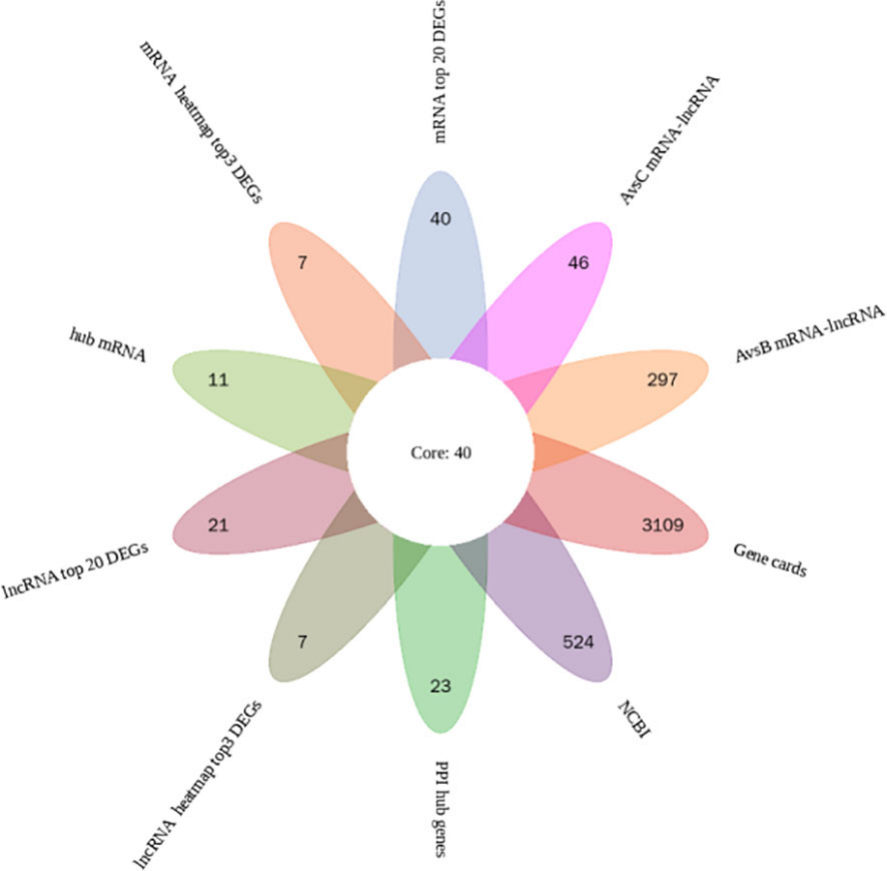
Multiple methods for screening hub genes.

The NCBI Gene database and GeneCards database were used to search for genes related to hepatitis B infection and immune regulation. A total of 3,108 genes were retrieved using the search terms “HBV infection OR immune regulation”. RT-qPCR validation was performed on 10 genes, including IFNAR1, JUN, KRAS, MAP2K6, E2F3, HRAS, MAPK12, ICAM1, NAMPT, and SOD2, selected based on the top 20 differentially expressed mRNAs and lncRNAs, top 3 clustering genes, key genes enriched in hepatitis B and immune-related pathways, and PPI network analysis, as well as intersection with retrieved genes (**Figure 11**).

### 3.5 RT-qPCR verification

To verify the reliability of transcriptomics, ten genes (IFNAR1, JUN, KRAS, MAP2K6, E2F3, HRAS, MAPK12, ICAM1, NAMPT, SOD2) were selected respectively according to their functional categories to detect whether their expression levels were consistent with those of the transcriptome by RT-qPCR. The RT qPCR results showed that compared with the group that successfully blocked HBV MTCT, the genes IFNAR1, MAP2K6, and SOD2 in the group that failed to block HBV MTCT were upregulated, while JUN, HRAS, and MAPK12 were downregulated. The sequencing results were consistent with qPCR validation. KRAS (log2FoldChange = 1.2724, t = -1.153) and E2F3 (log2FoldChange = 1.5550, t = -1.696) were up-regulated in the sequencing results but down-regulated in the qPCR verification. Compared with the healthy control group, ICAM1, NAMPT, and SOD2 were all up-regulated in the group with failed HBV MTCT blockage, and the sequencing results were consistent with the qPCR verification (**Table 3**).

**Table 3:** Results of RT-qPCR verification.

mRNA	mRNA_ log2FoldChange	mRNA_ regulation	lncRNA	lncRNA_ log2Fold Change	lncRNA_ regulation	qPCR-t	Group
IFNAR1	1.0750	Up	IL10RB-DT	2.0950	Up	0.354	AvsB
JUN	-1.0067	Down	ENSG00000231740	1.7805	Up	-1.441	
	-1.0067	Down	ENSG00000290013	-3.0490	Down		
KRAS	1.2724	Up	ENSG00000274987	3.4425	Up	-1.153	
MAP2K6	1.6927	Up	ENSG00000278972	1.3272	Up	0.151	
E2F3	1.5550	Up	ENSG00000227803	-1.2342	Down	-1.696	
HRAS	-1.2256	Down	ENSG00000288033	-1.1199	Down	-2.262	
MAPK12	-1.1459	Down	TRABD-AS1	-1.0880	Down	-0.933	
	-1.1459	Down	ENSG00000288871	-1.1345	Down		
SOD2	1.2996	Up	SOD2-OT1	5.5618	Up	1.638	
ICAM1	1.2250	Up	ENSG00000274425	1.0081	Up	3.466	AvsC
NAMPT	1.6324	Up	ENSG00000273320	2.1369	Up	3.408	
SOD2	1.2885	Up	SOD2-OT1	6.9378	Up	3.231	

There was a significant difference in the relative expression level of HRAS in peripheral blood between the group with failed HBV MTCT blockage and the group with successful HBV MTCT blockage, and HRAS was down-regulated in the group with failed HBV MTCT blockage (t = -2.262, FDR-adjusted p value < 0.05). The differences in the expression levels of ICAM1 (t = 3.466, FDR-adjusted p value = 0.006), NAMPT (t = 3.408, FDR-adjusted p value = 0.007), and SOD2 (t = 3.231, FDR-adjusted p value = 0.009) between the group with failed HBV MTCT blockage and the healthy control group were all statistically significant, and they were all up-regulated in the case group (**Table 4**).

**Table 4:** Statistical table of relative expression levels of target genes in samples relative to the reference gene GAPDH.

Sample	ICAM1	IFNAR1	JUN	KRAS	MAP2K6	E2F3	HRAS	MAPK12	NAMPT	SOD2
LB05	1.89%	39.69%	0.32%	14.02%	4.46%	9.72%	1.56%	0.01%	114.34%	53.84%
LB04	1.15%	32.23%	0.26%	13.06%	4.15%	8.38%	1.41%	0.01%	87.56%	32.84%
LB03	0.95%	28.62%	0.25%	11.41%	3.67%	6.86%	1.18%	0.01%	59.39%	13.95%
LB02	1.22%	24.71%	0.31%	11.16%	2.72%	8.46%	1.48%	0.02%	38.91%	10.11%
LB01	1.96%	41.47%	0.25%	17.76%	4.15%	7.64%	1.70%	0.02%	114.34%	48.30%
GB01	1.15%	39.59%	0.43%	16.57%	4.93%	8.13%	1.73%	0.06%	61.56%	22.32%
LA94	2.41%	37.63%	0.19%	18.26%	4.27%	10.15%	1.69%	0.07%	87.26%	54.59%
LA91	3.48%	53.10%	0.88%	27.23%	5.58%	14.56%	2.54%	0.02%	177.36%	75.79%
LA69	1.39%	39.32%	0.40%	15.35%	4.45%	10.20%	1.73%	0.03%	45.17%	18.13%
LA64	1.52%	42.73%	0.43%	16.84%	4.68%	11.32%	1.77%	0.03%	73.54%	29.39%
LA55	3.21%	53.84%	0.47%	24.74%	5.76%	12.24%	2.16%	0.03%	111.73%	61.84%
LA26	1.35%	24.94%	0.22%	12.91%	2.93%	6.46%	1.60%	0.02%	56.32%	13.62%
LA124	2.26%	27.11%	0.32%	11.56%	2.48%	6.75%	1.10%	0.03%	118.92%	59.87%
LA119	2.82%	40.33%	0.50%	16.34%	5.17%	12.27%	1.75%	0.02%	137.55%	52.97%
LA109	3.02%	44.08%	0.35%	19.77%	5.26%	10.10%	1.58%	0.04%	163.01%	65.98%
LA106	2.30%	38.25%	0.40%	16.96%	4.14%	10.83%	1.28%	0.03%	145.40%	66.13%
LC03	1.92%	46.38%	0.24%	12.26%	4.67%	7.48%	0.84%	0.03%	305.61%	74.48%
LC01	3.77%	47.30%	0.14%	10.54%	7.33%	7.28%	0.77%	0.01%	402.78%	103.77%
LA99	2.07%	34.35%	0.21%	15.59%	3.75%	7.07%	1.26%	0.03%	114.47%	48.69%
LA63	1.94%	35.36%	0.24%	18.26%	4.47%	10.08%	1.63%	0.02%	143.73%	39.32%
LA61	3.42%	47.63%	0.33%	19.43%	3.87%	10.91%	1.55%	0.02%	194.08%	66.28%
LA130	3.01%	38.96%	0.55%	16.15%	3.32%	8.60%	1.50%	0.04%	250.82%	81.23%
*FDR-adj p (*AvsC)	0.006	0.083	0.777	0.458	0.423	0.637	0.177	0.805	0.007	0.009
*FDR-adj p (*AvsB)	0.454	0.728	0.172	0.268	0.882	0.112	0.04	0.367	0.006	0.132

## 4 Discussion

According to previous studies, among children born to HBeAg-positive mothers, the failure rate of preventing HBV MTCT is approximately 5.8% when both hepatitis B vaccine and hepatitis B immunoglobulin are administered simultaneously, whereas the transmission rate increases to 10.3% when only the hepatitis B vaccine is used (24). Despite the effectiveness of current prophylactic strategies, a small proportion of infants still experience breakthrough infection, suggesting that additional host or viral factors may influence the failure of HBV MTCT prevention.

Previous studies on HBV MTCT have mainly focused on maternal viral factors and placental tissues. In contrast, PBMCs may reflect systemic immune responses associated with maternal–fetal immune interactions and neonatal antiviral immunity. Therefore, PBMC-based transcriptomic analysis may provide important insights into the immune mechanisms underlying HBV MTCT blockade failure.

In the present study, peripheral blood samples from infants with failed HBV MTCT blockade, infants with successful blockade, and healthy controls were analyzed using whole-transcriptome sequencing. By constructing lncRNA–mRNA co-expression networks and performing GO and KEGG enrichment analyses, this study aimed to identify immune-related transcriptomic characteristics associated with HBV MTCT outcomes.

HBV MTCT is a multifactorial process influenced by maternal viral load, HBeAg status, antiviral therapy, placental barrier function, and maternal–fetal immune interactions (25, 26). Even with standard passive-active immunoprophylaxis, a small proportion of infants still develop HBV infection, suggesting that host immune dysregulation and viral immune escape mechanisms may contribute to prophylaxis failure (27). Therefore, the findings of the present study should be interpreted as exploratory transcriptomic evidence rather than direct proof of causality.

Transcriptomic analysis revealed widespread alterations in immune-related gene expression patterns in the failed HBV MTCT blockade group compared with both the successful blockade group and healthy controls. Functional enrichment analyses demonstrated that these differentially expressed genes were mainly involved in antiviral immunity, cytokine-mediated signaling, T-cell activation, innate immune regulation, inflammatory responses, and phagosome-related pathways. These findings suggest that HBV MTCT blockade failure may be associated with dysregulated immune responses rather than isolated gene abnormalities.

The lncRNA sequencing results further supported the involvement of immune dysregulation in HBV MTCT blockade failure. Differentially expressed lncRNAs were mainly enriched in transcriptional regulation, cytokine signaling, apoptosis, autophagy, and immune-related biological processes. Increasing evidence suggests that lncRNAs play important roles in antiviral immunity through regulation of interferon signaling, inflammatory responses, immune-cell differentiation, and viral replication (28).

Notably, both mRNAs and lncRNAs were significantly enriched in the hepatitis B pathway when comparing the failed blockade group with the successful blockade group. Genes involved in this pathway included JUN, MAP2K6, KRAS, IFNAR1, E2F3, MAPK12, YWHAB, and HRAS. Among them, JUN occupied a central position in the PPI network, suggesting potential involvement in immune and inflammatory signaling regulation.

Previous studies have shown that JUN and MAPK-related signaling pathways participate in antiviral immune responses, cytokine regulation, and cellular stress signaling during HBV infection (29). IFNAR1 is a key receptor involved in type I interferon signaling and plays a critical role in innate antiviral immunity. Impaired IFNAR1 signaling may weaken antiviral immune responses and facilitate viral persistence (30).

In addition, MAPK and PI3K-Akt signaling pathways have been implicated in immune activation, inflammatory responses, and HBV-host interactions (31). Therefore, the enrichment of these immune-related pathways in the failed blockade group suggests that dysregulated antiviral immunity rather than tumor-associated mechanisms may contribute to HBV MTCT blockade failure.

These findings indicate that several genes identified in this study, including JUN, E2F3, KRAS, MAP2K6, and IFNAR1, are closely associated with liver disease and HBV-related pathological processes. Validation of these genes using qPCR suggests that they may be involved in the mechanisms underlying HBV MTCT. In addition, other differentially expressed genes identified in this study may serve as potential targets for future investigation.

RT-qPCR validation further showed that HRAS expression was significantly lower in the failed HBV MTCT blockade group compared with the successful blockade group, suggesting that downregulation of HRAS may be associated with transmission failure. In addition, ICAM1, NAMPT, and SOD2 were significantly upregulated in the failed blockade group compared with healthy controls. Compared with transcriptomic findings alone, these validated genes may represent more reliable candidate biomarkers associated with HBV MTCT blockade outcomes.

ICAM1 is an important adhesion molecule involved in leukocyte migration, inflammatory activation, and immune-cell interactions. Previous studies have shown that ICAM1 expression is elevated during HBV infection and may reflect ongoing immune activation and inflammatory responses (32, 33). Increased ICAM1 expression in the failed blockade group may therefore indicate enhanced inflammatory signaling and altered immune-cell trafficking during HBV exposure.

NAMPT is the rate-limiting enzyme in the NAD salvage pathway and plays an essential role in immunometabolic regulation. NAD metabolism critically influences T-cell activation, NK-cell function, and antiviral immune responses (34). Previous studies have suggested that NAMPT may promote HBV replication through regulation of cellular metabolism and immune signaling (35). Therefore, increased NAMPT expression observed in the present study may reflect altered immune metabolism associated with ineffective antiviral control during MTCT.

SOD2 is a key mitochondrial antioxidant enzyme involved in maintaining intracellular redox homeostasis. Oxidative stress has been reported to contribute to HBV-associated immune dysregulation and inflammatory injury (36). Increased SOD2 expression in the failed blockade group may therefore represent compensatory responses to oxidative stress induced by viral exposure and immune activation.

Importantly, these validated genes may have potential translational value. Future multicenter studies with larger independent cohorts are required to validate the reproducibility and predictive performance of ICAM1, NAMPT, SOD2, and HRAS. In addition, mechanistic studies using placental tissues, maternal PBMCs, cord blood, and functional immune assays are needed to clarify whether these genes directly contribute to impaired antiviral immunity and HBV MTCT blockade failure.

Taken together, the lncRNA–mRNA co-expression networks identified in this study suggest that these genes may participate in antiviral immune regulation, inflammatory responses, oxidative stress regulation, and maternal–fetal immune interactions during HBV MTCT. These findings provide a potential molecular basis for understanding the transcriptomic characteristics associated with MTCT outcomes of HBV MTCT and offer new targets for future research.

Several limitations of this study should be acknowledged. First, the sample size for whole-transcriptome sequencing was relatively small, particularly in the MTCT failure group, due to the low incidence of HBV prophylaxis failure in real-world cohorts. Small sample sizes may reduce statistical power and increase susceptibility to individual variability and false-positive findings. Previous studies have suggested that the statistical power of RNA-seq experiments depends not only on sample size but also on effect size, sequencing depth, and biological variability. For genes with relatively large fold changes, acceptable statistical power may still be achieved even with approximately five biological replicates per group (37). Nevertheless, small-sample transcriptomic studies should still be interpreted cautiously. Considering the relatively small sample size of this exploratory RNA-seq study, the statistical power after multiple-testing correction may have been limited. Although Benjamini–Hochberg FDR correction was applied to reduce false-positive findings, some potentially relevant genes may not have reached statistical significance because of limited statistical power. Taken together, the present study suggests that HBV MTCT blockade failure may be associated with dysregulated antiviral immunity, inflammatory activation, oxidative stress responses, and altered immune-metabolic regulation. The identified immune-related genes and lncRNAs provide potential molecular clues for understanding the immunological mechanisms underlying HBV MTCT and may serve as candidate biomarkers for future translational studies.

## 5 Conclusion

In this study, transcriptome sequencing was performed to characterize the differential expression profiles of mRNAs and lncRNAs among infants with failed HBV MTCT blockade, infants with successful MTCT blockade, and healthy controls. RT-qPCR validation further demonstrated that downregulation of HRAS may be associated with the failure of HBV MTCT prevention, whereas upregulation of NAMPT, ICAM1, and SOD2 may be related to HBV MTCT.

GO and KEGG enrichment analyses indicated that the differentially expressed genes were mainly involved in immune-related pathways, including cytokine-mediated signaling, innate immune responses, and immune processes involving neutrophils and T cells, as well as pathways such as the NOD-like receptor signaling pathway and the hepatitis B pathway.

Overall, this study provides preliminary evidence that dysregulated lncRNA–mRNA transcriptional networks may be associated with HBV MTCT. These findings offer new insights into the molecular mechanisms underlying HBV MTCT and may provide potential biomarkers and targets for improving strategies for preventing vertical transmission of HBV. However, further studies with larger sample sizes and functional experiments are required to validate these findings and clarify the underlying mechanisms.

## Data Availability

The datasets presented in this article are not readily available because the RNA-sequencing data contain human genetic resource information derived from infant participants and are subject to the Regulations of the People’s Republic of China on the Administration of Human Genetic Resources. The datasets have been deposited in the National Genomics Data Center (NGDC) Open Archive for Miscellaneous Data (OMIX) under accession number OMIX017540 and are available through controlled access. Requests to access the datasets should be directed to the NGDC OMIX repository (https://ngdc.cncb.ac.cn/omix/) and will be considered in accordance with applicable legal, ethical, and institutional requirements.
